# Feasibility of MRI for the evaluation of interosseous ligament vertical segment via subtalar arthroscopy correlation: comparison of 2D and 3D MR images

**DOI:** 10.1186/s12891-021-04759-8

**Published:** 2021-10-12

**Authors:** Hong-Geun Jung, Sung Gyu Moon, Deuk Young Yoon, Hyemin Jang, Ji Hee Kang

**Affiliations:** 1 Department of Orthopedic Surgery, Konkuk University Medical Center, Konkuk University School of Medicine, Gwangjin-gu, Seoul, South Korea; 2 Department of Radiology, Konkuk University Medical Center, Konkuk University School of Medicine, 120-1 Neungdong-ro, Gwangjin-gu, Seoul, 05030 South Korea

**Keywords:** Instability, Subtalar joint, Interosseous ligament, Ankle, MRI

## Abstract

**Background:**

Interosseous ligament vertical segment (IOLV) and calcaneofibular ligament (CFL) have been reported to be important in stabilizing the subtalar joint. Unlike CFL, there is not much information regarding the comparison of MRI results with surgical evaluation of IOLV and the comparison between 2D and 3D MRI on IOLV evaluation. The feasibility of MRI in IOLV evaluation has yet to be reported. The purpose of this study was to evaluate the validity and reliability of MRI in IOLV tear detection via correlation with arthroscopic results. We also compared the diagnostic performance of 2D and 3D MR images.

**Methods:**

In this retrospective study, 52 patients who underwent subtalar arthroscopy after ankle MRI were enrolled. Arthroscopic results confirmed IOLV tear in 25 cases and intact IOLV in 27 cases. Two radiologists independently evaluated the IOLV tears using only conventional 2D images, followed by isotropic 3D images, and comparison with arthroscopic results.

**Results:**

Only the 2D sequences interpreted by two readers showed a sensitivity of 64.0–96.0%, a specificity of 29.6–44.4%, a positive predictive value of 51.6–56.4%, and a negative predictive value of 57.1–88.9%. Addition of isotropic 3D sequences changed the sensitivity to 60.0–80.0%, specificity to 63.0–77.8%, positive predictive value to 64.3–76.9%, and negative predictive value to 66.7–80.8%. The overall diagnostic performance of isotropic 3D sequences (AUC values: 0.679–0.816) was higher than that of 2D sequences (AUC values: 0.568–0.647). Inter-observer and intra-observer agreement between the two readers was moderate-to-good for both 2D and 3D sequences. The diagnostic accuracy in 19 patients with tarsal sinus fat obliteration tended to increase from 26.3–42.1% to 57.9–73.7% with isotropic 3D sequences compared with 2D sequences.

**Conclusions:**

Isotropic 3D MRI was feasible for the assessment of IOLV tear prior to subtalar arthroscopy. Additional 3D sequences showed higher diagnostic accuracy compared with conventional 2D sequences in IOLV evaluation. Isotropic 3D sequences may be more valuable in detecting IOLV tear in case of tarsal sinus fat obliteration.

## Background

Subtalar instability (STI) is characterized by anterior or medial shift as well as varus tilt of the calcaneus beneath the talus [1, 2]. STI can be surgically established by remarkable widening of subtalar joint and medial translation of the calcaneus to the talus via intraoperative stress fluoroscopy under anesthesia [3, 4]. STI and lateral ankle instability (LAI) exhibit similar injury mechanism and symptoms, and therefore it is difficult to distinguish the two disorders clinically [5–7]. Furthermore, STI can develop in an isolated form, but can also occur in conjunction with LAI [8, 9]. Since the two disorders (STI and LAI) are treated differently, an accurate preoperative diagnosis is required. Although radiographic findings and validated imaging techniques for LAI are well known, imaging findings of STI have yet to be reported. Effective imaging techniques have yet to be identified. Stress radiographs are generally used but their reliability is still disputed [2, 10, 11]. Clinical diagnosis may depend on several parameters including MRI for the assessment of the morphology of ligamentous structure and associated lesions underlying the disorder [11].

**Fig. 1:**
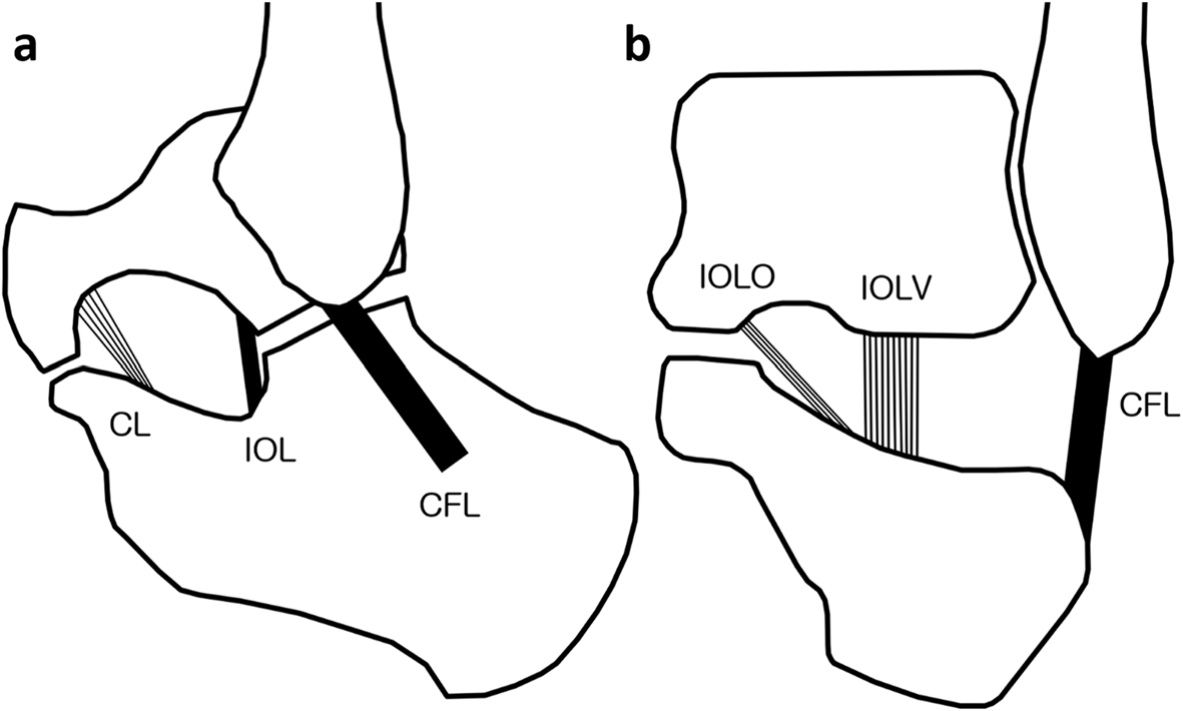
Ligaments around the tarsal sinus. **a** In the sagittal view, CL is located in the anterior aspect of the tarsal sinus and extends from talus neck to calcaneus neck. IOL is located immediately anterior to the subtalar joint. CFL is located on the lateral side of the subtalar joint and crosses both the ankle and the subtalar joint. **b** In the coronal view, IOL contains two segments with distinct insertion and running patterns. IOLO is located further inside the tarsal sinus and traverses obliquely from the talus in the tarsal canal to the calcaneus. IOLV is oriented vertically, located in the center of tarsal sinus and further outward than the IOLO. CL, cervical ligament; IOL, interosseous ligament; CFL, calcaneofibular ligament; IOLO, interosseous ligament oblique segment; IOLV, interosseous ligament vertical segment

Previous anatomic studies suggest that interosseous ligament, cervical ligament, inferior extensor retinaculum, and calcaneofibular ligament (CFL) may stabilize the subtalar joint [12, 13]. In addition, interosseous ligament contains two distinct ligaments with characteristic insertion and running patterns: anterior capsular ligament (or interosseous ligament vertical segment, IOLV) and interosseous talocalcaneal ligament (or interosseous ligament oblique segment, IOLO) [14] (Fig. 1). IOLV is known to originate at the anterior margin of the posterior facet of the talus, extending vertically across the subtalar joint and attach to the calcaneus [3, 15]. In contrast, IOLO runs diagonally from the talus in the tarsal canal to the calcaneus. IOLV is centrally located close to the posterior talocalcaneal joint, while IOLO is located inside the tarsal sinus [3]. Recent studies have indicated that IOLV and CFL are important stabilizers of subtalar joint [3, 15, 16]. Comparing patients with STI or LAI with normal individuals, no significant differences in abnormalities of cervical ligament, inferior extensor retinaculum, or IOLO were detected [3, 15]. CFL abnormalities were detected only when compared with normal controls [15], and did not differ between STI and LAI patients [3]. These studies suggested that IOLV may play a critical role as an anterior-medial stabilizer of the posterior subtalar joint, whereas CFL may play a role in posterior-lateral stabilization of the subtalar joint.

MRI has been widely used to evaluate ankle collateral ligaments including CFL [17, 18]. Unlike CFL, there is not much information available regarding the comparison between surgical evaluation and MRI results for IOLV injury. If MRI can be used to predictably assess the condition of IOLV, it can be used to determine the surgical procedure for patients with STI. Three-dimensional isotropic MRI is an alternative imaging tool for the evaluation of subtalar ligaments. It provides a detailed description of morphological findings as it can reduce the partial volume averaging effect based on thinner sections and multi-planar reformation in arbitrary planes [19]. To the best of our knowledge, the feasibility of MRI in IOLV evaluation has yet to be reported. No comparative studies of 2D and 3D MRI for IOLV evaluation have been published. The correlation between MRI and surgery is also unknown. Therefore, the purpose of this study is to retrospectively investigate preoperative MRI and its validity and reliability in IOLV evaluation based on correlation with arthroscopic results. We compared the diagnostic performance of 2D conventional images and additional 3D isotropic images via 3 T MRI. We hypothesized that the diagnostic performance of 2D images does not surpass that of 3D isotropic images, and 3D isotropic MRI is a feasible option to assess the presence of IOLV tear prior to subtalar arthroscopy.

## Methods

### Study population

This study was designed as a retrospective observational study based on image and chart review. The institutional Review Board of our hospital approved this study and also waived the need for informed consent due to its retrospective nature. A total of 136 patients underwent ankle MRI and subtalar arthroscopy in our hospital from January 2012 to January 2018. The study enrolled a total of 52 patients based on the following inclusion criteria: (a) preoperative MRI performed at our institution; (b) single MR device with standardized protocol; (c) subtalar arthroscopy performed less than 6 months after MRI; and (d) no history of prior subtalar joint surgery. A total of 84 patients were excluded, including 47 patients who underwent preoperative MRI at an external institution, 26 patients undergoing MRI using different devices at our institution, including one patient with no available 3D image, nine who did not undergo surgery within 6 months after MRI, and one with a history of prior subtalar joint surgery. The 52 patients included 24 females and 28 males. Their mean age was 34.4 years (range, 15 to 75 years). The analysis involved 24 right ankles and 28 left ankles. The mean interval between MRI and arthroscopy was 66.4 days (range, 0–181 days).

Of the 52 ankles, 12 had isolated STI, and 15 showed a combination of STI and LAI. Eleven ankles exhibited isolated LAI. Six patients had post-traumatic soft tissue impingement. Synovitis associated with osteochondral lesions of the talus was found in 3 cases, rheumatoid arthritis in 2 cases, posterior tibial tendinopathy in 1 case, spring ligament rupture in 1 case, and localized synovial hyperplasia in 1 case. Twenty-nine of these fifty-two patients were clinically diagnosed with sinus tarsi syndrome before surgery.

The mean duration of symptoms was 4.2 years (range, 1–14 years). Most patients, including 38 with STI or LAI, had a prior history of ankle sprain and symptomatic recurrent ankle sprain prior to surgery. Subtalar arthroscopy was performed in patients with persistent symptoms despite conservative treatment for more than 6 months.

### Arthroscopy

Subtalar arthroscopy was provided to patients with suspected STI or resistant sinus tarsi syndrome. The diagnostic criteria for STI were established based on comprehensive clinical, radiographic, and arthroscopic factors. Clinical diagnosis of STI was based on the presence of at least 4 of the 5 following preoperative criteria [4, 20]: (1) recurrent ankle sprain, (2) tarsal sinus pain and tenderness, (3) hind foot looseness or giving way, (4) hind foot instability on physical examination, and (5) radiographic STI on Broden’s varus stress views (ipsilateral subtalar tilt > 10° or contralateral subtalar tilt difference > 5°). Surgical diagnosis of STI was established by significant subtalar joint widening (subtalar tilt > 10°) or medial calcaneal translation (> 5 mm) on C-arm stress fluoroscopy under anesthesia, in addition to chronic IOLV tear on subtalar arthroscopy. Sinus tarsi syndrome was clinically diagnosed based on pain elicited by palpation of the sinus tarsi, exacerbation by foot inversion/eversion, and pain cessation following injection of local anesthetics into the sinus tarsi [21].

All arthroscopies were provided by a senior orthopedic surgeon in our institution. Ankle and subtalar arthroscopies were routinely performed prior to index surgery to assess joint pathology. Ankle joint was examined using standard anterolateral and anteromedial portals under distracted condition. Synovectomy was performed for synovitis. After removal of distraction, subtalar joint was evaluated using central and anterolateral portals [4]. Subtalar arthroscopy was performed to investigate subtalar joint laxity, chronic tear of the IOLV, synovitis in the tarsal sinus, and other features.

Arthroscopic findings were reviewed retrospectively based on surgical records with standardized evaluation of the IOLV condition (e.g., pathway, tautness, continuity, tissue quality, and adhesions). Based on arthroscopic findings as a standard of reference, patients were divided into two groups: IOLV tear and IOLV intact. Intact IOLV refers to preserved continuity and tautness of the ligament. Ligament dysfunction due to chronic IOLV tear was defined by one or more of the following diagnostic criteria: abnormal pathways, loss of tautness, distinct discontinuity with a defect filled by fibrous tissues, and adhesion of surrounding tissues.

### MRI protocol

MR studies were performed using a 3-T imaging system (Magnetom Skyra, Siemens Healthcare, Erlangen, Germany). Patients were examined in a supine position with the ankle in a neutral position using a phased-array foot-and-ankle coil with 16 channels. Our institutional protocol for ankle MRI with this system included the following 2D turbo spin-echo sequences: axial and coronal T2-, sagittal T1-, sagittal T2- with fat suppression, and axial, coronal, sagittal T1- weighted images with contrast enhancement. In addition to our routine 2D sequence, 3D imaging data were obtained along the sagittal plane using T2-weighted 3D isotropic turbo spin-echo sequence without fat suppression. Parameters used for the 3D sequence were: repetition time, 1200 ms; echo time, 158 ms; flip angle, 120°; echo train length, 68; bandwidth, 362 kHz/pixel; field of view, 140 mm; matrix, 256 × 230; and section thickness, 0.5 mm. The 3D sequences were subsequently reformatted into orthogonal axial and coronal plane with a slice thickness of 0.6 mm without inter-slice gap by technicians using commercially available software programs (Syngo MR, Siemens Healthcare). Specific parameters used for each sequence are summarized in Table 1.

**Table 1:** Imaging parameters for ankle MRI

Parameters	2D Conventional				3D Isotropic
	Sag T2 FS	Ax T2	Cor T2	Sag T1	3D Sag T2
TR/TE (ms)	3160/48	4770/76	5070/76	704/13	1200/158
Flip angle (°)	150	150	150	150	120
Matrix size	384*250	512*229	512*211	512*333	256*230
Field of view (cm)	14	14	14	14	14
Section thickness (mm)	3	3	3	3	0.5
Intersection gap (mm)	0	0	0	0	0
Bandwidth (kHz/pixel)	151	199	199	250	362
Echo train length	7	11	11	3	68
No. acquisition	1	1	1	1	1
Scan time	3:54	3:25	3:23	2:43	6:45

### Image analysis

Two musculoskeletal radiologists with 18 and 3 years of experience, respectively, analyzed these images. Image evaluation of IOLV was conducted via two methods. First, each reader independently evaluated the IOLV using routine 2D MR image set alone (method 1). Then, a combination of 2D and 3D isotropic image sets were used (method 2). Both readers were blinded to arthroscopic results and to each other’s analyses. The second review session was conducted similarly at a three-month interval blinded to the results of previous analysis.

**Fig. 2:**
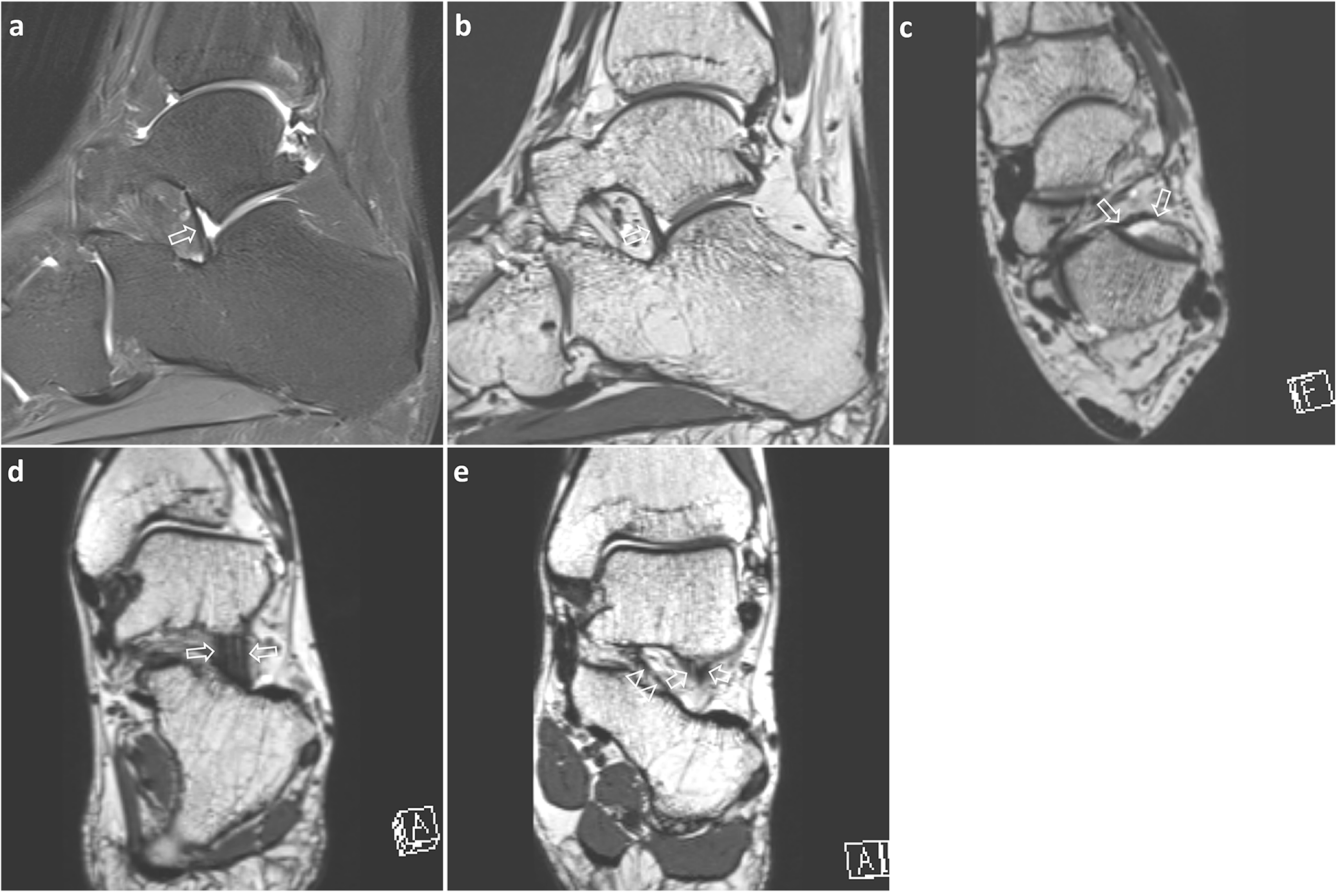
A 29-year-old man with posttraumatic soft tissue functional impingement. Sagittal T2-weighted image with fat suppression (**a**) demonstrates uniform thickness of band-shaped IOLV (arrow) before posterior talocalcaneal joint. Sagittal (**b**), axial (**c**) and oblique coronal (**d**) 3D isotropic T2-weighted images represent IOLV thickness and width (arrows), starting from the anterior margin of the posterior facet of the talus and extending vertically across the subtalar joint. In the coronal (**e**) 3D isotropic image along the posterior wall of the tarsal sinus, IOLO (arrowheads) courses obliquely from the inside, and IOLV (arrows, partially visualized) runs vertically further outward. IOLV, interosseous ligament vertical segment; IOLO, interosseous ligament oblique segment

**Fig. 3:**
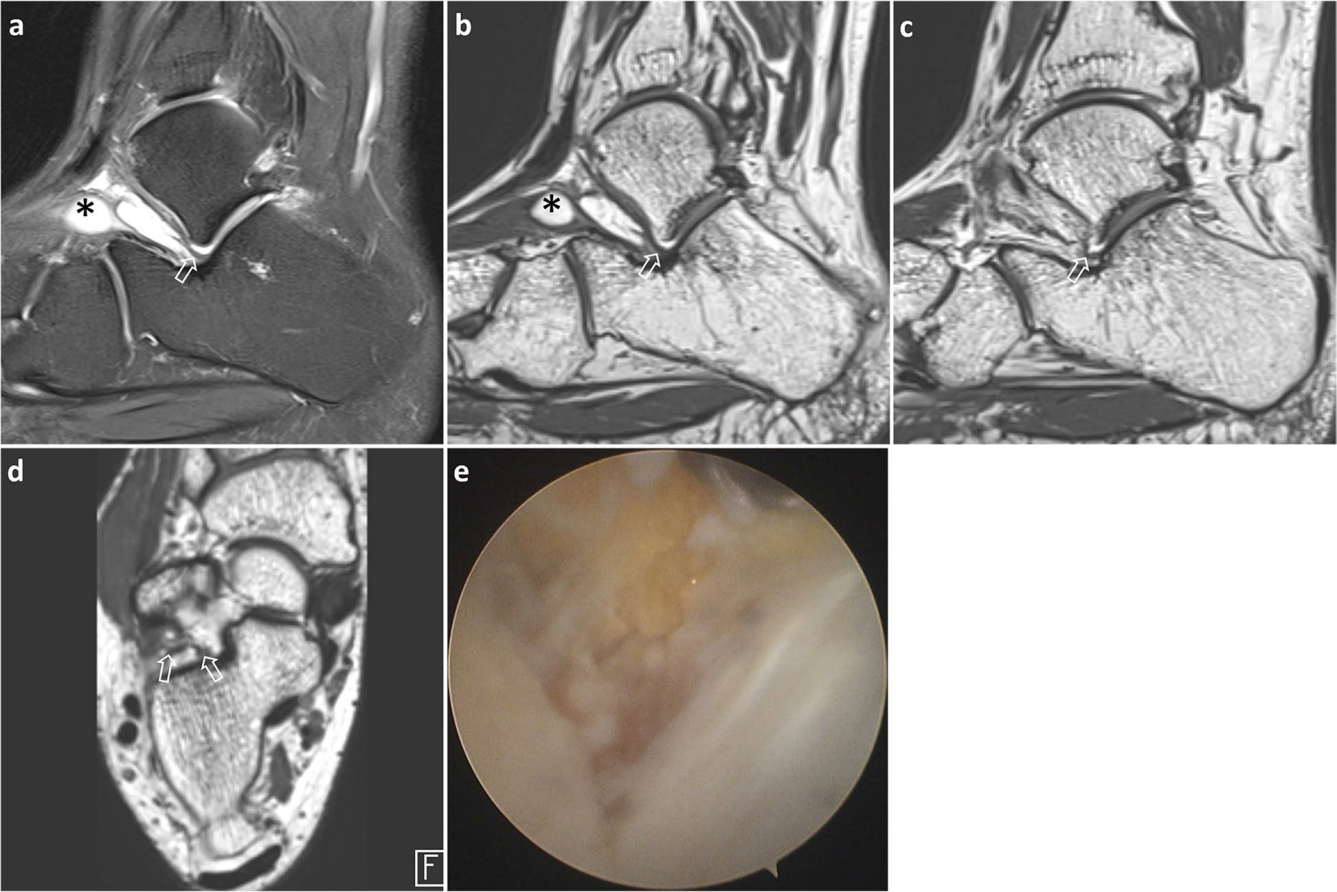
A 31-year-old man with subtalar instability. Sagittal T2-weighted image (**a**) shows normal-looking IOLV (arrow) with uniform thickness and continuity in tarsal sinus. Ganglion cysts (*) along inferior extensor retinaculum induce tarsal sinus fat obliteration. 3D isotropic sagittal images (**b** and **c**) acquired sequentially in the medial direction and axial image (**d**) demonstrate irregularity in the inner half of IOLV (arrows). Arthroscopic image (**e**) of the tarsal sinus reveals the talocalcaneal joint on the right side and a chronic IOLV tear in the inner half; IOLV, interosseous ligament vertical segment

IOLV status was graded using a 3-point system: grade 0, intact ligament; grade 1, partial tear; and grade 2, complete tear. Normally, IOLV is defined as the thick part of the anterior capsule of the posterior talocalcaneal joint (Fig. 2). It is attached to the calcaneus, starting at the anterior edge of the posterior facet of the talus and vertically crossing the anterior side of the subtalar joint [14]. Based on T2-weighted images, intact ligaments are defined as homogeneous, low signal intensity ligaments with uniform thickness, width, and normal pathway [3]. Ligament tear is defined by partially or entirely invisible ligaments due to discontinuities, abnormal pathways, irregularities, or an inhomogeneous signal increase in the ligament [15] (Fig. 3). Grade 2 injury is characterized by non-visualization, complete interruption, and highly irregular thin appearance suggesting scar tissue. To assess the diagnostic validity of these two methods, the IOLV status was divided into two groups: IOLV tear and IOLV intact. Grade 1 and grade 2 injuries were classified as IOLV tear while grade 0 was classified as IOLV intact. MRI findings were correlated with arthroscopic results as a standard of reference.

**Fig. 4:**
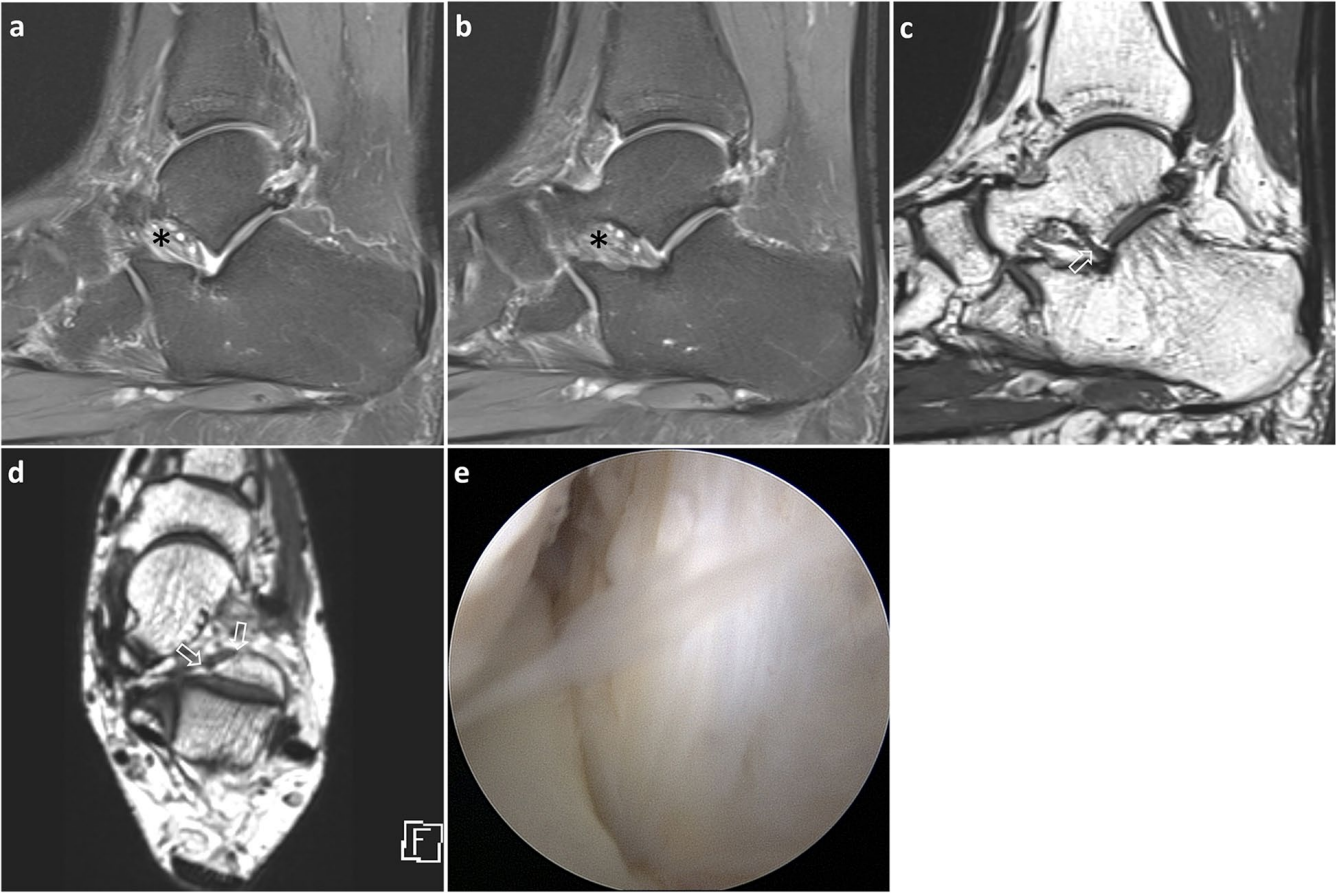
A 54-year-old woman with sinus tarsi syndrome. Sagittal images (**a** and **b**) obtained sequentially in the medial direction indicate an invisible IOLV with tarsal sinus fat edema (*). 3D isotropic sagittal (**c**) and axial (**d**) images show a normal IOLV (arrows) with uniform thickness and continuity before posterior talocalcaneal joint. Arthroscopic image (**e**) of the tarsal sinus reveals the talocalcaneal joint on the right side and a normal IOLV; IOLV, interosseous ligament vertical segment

In addition, edema or obliteration of tarsal sinus fat, which is frequently associated with sinus tarsi syndrome and STI [22], was evaluated using fat suppressed sagittal T2-weighted and sagittal T1-weighted images with and without contrast enhancement (Fig. 4). Diagnostic accuracy between patients with and without tarsal sinus fat abnormality was assessed for each method and reader in different sessions.

In quantitative analysis, IOLV dimensions were measured on a 4x magnified image of the plane that best represented the structure, excluding patients whose IOLV was not visualized or discontinuous. Thickness and width were measured at the mid-portion of the ligament on sagittal and axial isotropic 3D T2-weighted images, respectively. Measurements were acquired three times by one investigator. The mean values were recorded in millimeters. All measurements were performed using the measurement tool built into GE PACS software (GE Healthcare, Mt. Prospect, IL, USA).

As an ancillary finding, MRI findings of CFL were investigated in 38 patients diagnosed with STI or LAI who underwent CFL reconstruction or repair for treatment. CFL status were assessed and designated as normal or injured. MRI findings of injured CFL were defined when the ligament was partially or entirely invisible due to discontinuity, abnormal pathway, irregularity, or inhomogeneous signal increase or enhancement in the ligament. They were evaluated by consensus of two readers using a combination of 2D and 3D imaging sequences.

### Statistical analysis

Continuous data were analyzed with Mann-Whitney test. The diagnostic performance of each method in IOLV tear detection was evaluated in terms of sensitivity, specificity, positive and negative predictive values based on binominal distribution. To compare the diagnostic accuracies of the two methods, the receiver operating characteristic curve (ROC) and area under the curve (AUC) were determined by classifying the IOLV as intact or tear based on MRI grade. The difference in AUC values between the two methods was calculated using the DeLong test. Inter-observer and intra-observer agreements were calculated for each method using Kappa statistics. Kappa values were interpreted as follows: 0.01–0.20, slight; 0.21–0.40, fair; 0.41–0.60, moderate; 0.61–0.80, good; and 0.81–1.00, excellent. ROC analysis was used to determine the cutoff value of the IOLV dimension to differentiate between IOLV intact and IOLV tear. *P* values less than 0.05 indicated statistical significance. All statistical analyses were performed using SPSS version 25 (IBM Corp., Armonk, NY, USA) or MedCalc version 16.2.1 (MedCalc Software, Ostend, Belgium).

## Results

### Group characteristics

Arthroscopic results confirmed IOLV tear in 25 of 52 patients and intact IOLV in 27 patients. STI was diagnosed in 23 of 25 patients with IOLV tear. However, 4 out of 27 patients with intact IOLV showed STI. Of the 27 patients with STI, 23 had IOLV tear. Arthroscopic examination of all patients with IOLV tears showed fibrous or hypertrophied synovium in the subtalar joint. There was no significant difference in age (*p* = 0.343), sex distribution (*p* = 0.419), right/left (*p* = 0.266), tarsal sinus tenderness on physical examination (*p* = 0.245), or obliteration of tarsal sinus fat on MRI (*p* = 0.259) between the two groups (Table 2).

**Table 2:** Group characteristics

	IOLV intact	IOLV tear	*P*-value
Age	36.2 ± 16.5	32.4 ± 11.4	0.343
Sex (M:F)	13:14	15:10	0.419
Right:Left	10:17	14:11	0.266
Tarsal sinus tenderness	11/27 (40.7%)	6/25 (24.0%)	0.245
Tarsal sinus fat obliteration	12/27 (44.4%)	7/25 (28.0%)	0.259

Note. Data are presented as number of patients (percent). P values were determined using the Fisher’s exact test

In 38 patients diagnosed with STI or LAI who underwent CFL reconstruction or repair, MRI showed normal CFL in 8 patients and CFL injury was observed in 30 patients. CFL injury was found in 8 of 12 isolated STIs, 14 of 15 combined with STI and LAI, and 8 of 11 isolated LAIs. Overall 78.9% of patients with STI or LAI exhibited CFL injury.

### Diagnostic performance according to the evaluation methods

For each reader and each session, diagnostic validities of the two methods are summarized in Table 3. In each session, both readers found that method 2 had greater specificity and positive predictive value for diagnosing IOLV tear than method 1, although the sensitivity and negative predictive values varied depending on the reader or session.

**Table 3:** Diagnostic validity of each method for IOLV tears

	Sensitivity		Specificity		PPV		NPV	
	Reader 1	Reader 2	Reader 1	Reader 2	Reader 1	Reader 2	Reader 1	Reader 2
1st session								
Method 1	64.0 (16/25)	80.0 (20/25)	44.4 (12/27)	33.3 (9/27)	51.6 (16/31)	55.3 (21/38)	57.1 (12/21)	71.4 (10/14)
Method 2	60.0 (15/25)	80.0 (20/25)	74.1 (20/27)	77.8 (21/27)	68.2 (15/22)	76.9 (20/26)	66.7 (20/30)	80.8 (21/26)
2nd session								
Method 1	96.0 (24/25)	88.0 (22/25)	29.6 (8/27)	37.0 (10/27)	55.8 (24/43)	56.4 (22/39)	88.9 (8/9)	76.9 (10/13)
Method 2	72.0 (18/25)	76.0 (19/25)	63.0 (17/27)	70.4 (19/27)	64.3 (18/28)	70.4 (19/27)	70.8 (17/24)	76.0 (19/25)

Note. Data are presented as percentages, with number of patients in parentheses

*PPV* positive predictive value, *NPV* negative predictive value

Table 4 summarizes AUC values indicating the diagnostic accuracy of IOLV interpretation for each reader and each method in each session. For both readers, the overall diagnostic performance was higher with method 2 (AUC values: 0.679–0.816) than with method 1 (AUC values: 0.568–0.647), although the difference in diagnostic accuracy was statistically significant only for reader 2 in the first session (*p* = 0.016) and not significant for the remaining sessions (*p* = 0.099–0.299).

**Table 4:** AUC values for the accuracy of IOLV evaluation for each reader and method in different sessions

	AUC	SE	95% CI	*P* value
1st session				
Reader 1				
Method 1	0.568	0.075	0.423–0.705	
Method 2	0.679	0.067	0.535–0.802	
Difference	0.111	0.076	−0.038-0.261	0.145
Reader 2				
Method 1	0.636	0.071	0.491–0.765	
Method 2	0.816	0.054	0.684–0.909	
Difference	0.180	0.075	0.033–0.327	0.016
2nd session				
Reader 1				
Method 1	0.633	0.072	0.488–0.763	
Method 2	0.700	0.068	0.557–0.819	
Difference	0.067	0.064	−0.059-0.193	0.299
Reader 2				
Method 1	0.647	0.07	0.503–0.779	
Method 2	0.753	0.063	0.614–0.862	
Difference	0.106	0.064	−0.020-0.232	0.099

Note. Data represent AUC values, standard errors, 95% confidence intervals for the diagnostic performance of each method, classified as intact or tear based on MRI grade using ROC curves. *P*-value represents the statistical significance of the differences between the two AUCs using methods 1 and 2, calculated using the DeLong test

For method 1, the inter-observer agreement was good (κ = 0.620, 0.771 for each session) and the intra-observer agreement was moderate-to-good (κ = 0.472, 0.750 for each reader). For method 2, the inter-observer agreement was moderate-to-good (κ = 0.538, 0.653 for each session) and the intra-observer agreement was moderate-to-good (κ = 0.620, 0.500 for each reader). Both readers found that methods 1 and 2 had moderate or higher inter-observer and intra-observer agreements to assess the status of IOLV.

### Diagnostic accuracy between patients with and without tarsal sinus fat abnormality

Method 2 had higher specificity and positive predictive value than method 1 for the overall detection of IOLV tear. Misinterpretations generated by method 1 were correctly interpreted using method 2 in 7 and 10 cases by the two readers, respectively, in the first session (Fig. 3). In the second session, 6 and 5 cases were correctly interpreted using method 2 by the two readers, respectively.

In the first session involving 19 patients with tarsal sinus fat abnormality, reader 1 correctly interpreted five cases of intact IOLV and reader 2 correctly interpreted eight cases of intact IOLV. In the second session, each of the two readers correctly interpreted six and four cases of intact IOLV using method 2. The diagnostic accuracy of readers A and B was 57.9–63.2% and 63.2–73.7%, respectively, with method 2, and 31.6–36.8% and 26.3–42.1%, respectively, with method 1 (Table 5, Fig. 4). In cases involving tarsal sinus fat edema or obliteration, the diagnostic accuracy tended to increase with isotropic 3D sequences.

**Table 5:** Diagnostic accuracy between patients with and without tarsal sinus fat abnormality

	Tarsal sinus fat abnormality (+)		Tarsal sinus fat abnormality (−)	
	Method 1	Method 2	Method 1	Method 2
1st session				
Reader 1	6/19 (31.6)	11/19 (57.9)	22/33 (66.7)	24/33 (72.7)
Reader 2	5/19 (26.3)	14/19 (73.7)	24/33 (72.7)	27/33 (81.8)
2nd session				
Reader 1	7/19 (36.8)	12/19 (63.2)	26/33 (78.8)	22/33 (66.7)
Reader 2	8/19 (42.1)	12/19 (63.2)	25/33 (75.8)	25/33 (75.8)

Note. Data are presented as number of patients (percent)

Conversely, in 33 cases without tarsal sinus fat abnormality, the diagnostic accuracy of readers A and B was 66.7–78.8% and 72.7–75.8%, respectively, using method 1, and 66.7–72.7% and 75.8–81.8%, respectively, using method 1 (Table 5). Therefore, the difference between methods 1 and 2 was negligible in cases without tarsal sinus fat edema or obliteration.

### IOLV dimensions using 3D isotropic image

With the exception of 15 patients whose IOLV was not visualized or discontinuous, the group with IOLV tears had significantly smaller width than the group with intact IOLVs (6.37 ± 1.59 vs. 7.90 ± 1.32 mm, *p* = 0.003, Mann-Whitney test). However, ligament thickness of the IOLV tear group did not differ significantly from that of the intact IOLV group (1.97 ± 0.66 vs. 2.10 ± 0.55 mm, *p* = 0.538). ROC analysis of IOLV width showed that a cutoff width of 7.50 mm was associated with a sensitivity of 76.9% and a specificity of 75.0% (AUC = 0.801; *p* = 0.003) for distinguishing IOLV tears from intact IOLVs.

## Discussion

The principal finding of the present study is that 3D isotropic MRI is superior to 2D conventional MRI in predicting IOLV tears prior to subtalar arthroscopy. The diagnostic accuracy of isotropic 3D sequences tends to increase, especially for IOLV evaluation in the presence of tarsal sinus fat abnormality. Therefore, the isotropic 3D sequences are useful in evaluating ankles with suspected STI or sinus tarsi syndrome. Quantitatively, the IOLV dimension was significantly smaller in width in the group with IOLV tears than in intact IOLV group.

Because subtalar disorders are less known, the structures in the tarsal sinus may be overlooked [10]. It may be attributed to limited consensus about the definition of anatomic structures and the interpretation of their biomechanical relevance in tarsal sinus [16]. Repetitive micro-trauma of the interosseous and neighboring ligaments can contribute to chronic ligamentous attenuation and result in instability of consecutive ligamentous components [2]. Efforts have been made to evaluate these ligamentous lesions via noninvasive imaging analysis using stress radiograph, CT, or MRI [15, 23, 24]. High-resolution MRI of the ankle and subtalar joint facilitates the identification and differentiation of the ligament injury prior to surgical reconstruction. Intraoperative stress fluoroscopy and subtalar arthroscopy can be performed to visualize subtalar joint laxity and synovitis and diagnose associated ligamentous lesions [4, 25]. Recent MRI studies have indicated that IOLV in tarsal sinus and CFL are important stabilizers of the subtalar joint [3, 15]. According to a previous study using 3D isotropic imaging, tears involving IOLV and CFL are significantly more frequent in the STI group than in the normal group [15]. Another study comparing isolated STI group and isolated LAI group found that IOLV tear was more common in isolated STI group, while there is no significant difference in CFL tear between the two groups [3]. Accordingly, it could be inferred that CFL tear is related to both STI and LAI, while IOLV tear appears to be directly related to STI. CFL, located lateral to the subtalar joint, is the only component of the collateral ligament that crosses both the ankle and the subtalar joint. CFL essentially contributes to LAI and STI in case of injury [7, 26]. IOLV located in front of the subtalar joint stabilizes the subtalar joint anteriorly, implying that IOLV tear is associated with STI.

Comparative studies evaluating the subtalar ligaments using MRI have been reported using only 3D images [3, 15]. However, many institutions still utilize only 2D images for ankle MRI. Therefore, it is unclear whether the results based on 3D images can be used in conventional 2D images. Our study was the first to compare 2D images with additional 3D isotropic images for the evaluation of subtalar ligaments. We hypothesized that the diagnostic performance of 2D image does not surpass that of 3D isotropic sequence because of the thick slices used for thin structures like IOLV. The diagnostic performance of 2D (method 1) and 3D isotropic sequences (method 2) was comparable. Our results showed that the addition of 3D sequences (method 2) improved the diagnostic performance of 2D sequences alone (method 1) in the evaluation of IOLV injury. Although not all reviewers found significant differences in AUC value between the two methods, the AUC values tended to increase from method 1 to method 2.

STI was frequently associated with sinus tarsi syndrome. Sinus tarsi syndrome has been linked to scarring of the interosseous ligament on arthroscopy and sinus fat edema or fat obliteration on MRI, which makes it difficult to assess sinus structure [21, 27]. Notably, in 19 cases diagnosed with tarsal sinus fat abnormalities, the diagnostic accuracy tended to increase with method 2, despite the lack of statistical evidence due to the small number of cases. Using method 2, each reader correctly interpreted the IOLVs that were incorrectly rated with method 1. The present study demonstrates that while conventional 2D images have obvious limitations when evaluating small structures such as IOLV, 3D images represent a feasible and more accurate alternative. We believe that adding 3D images to the routine ankle MR protocol is appropriate in conditions where STI or refractory sinus tarsi syndrome is clinically suspected.

There are several reasons why 3D imaging should be included in the ankle MR protocol. Using slices thicker than 3 mm in 2D sagittal sequence can result in partial volume average that obscures the IOLV. The 3D isotropic sequence provides a detailed view of ligaments with a slice thickness of 1 mm or less and allows multi-planar reconstruction of the ankle lateral ligaments [19, 28]. The reconstructed MR plane can be used to simultaneously visualize the width and length of the entire ligament. However, the blurring phenomenon in the 3D image contributes to the overestimation or underestimation of ligament tear. Poor image sharpness and low structural edge discrimination are other limitations of 3D isotropic sequences due to low bandwidth usage and loss of uniformity between planes [18, 29].

Of 25 patients with IOLV tear, 92% had STI. However, 14.8% of 27 intact IOLVs showed STI. In this study, ligament tear was defined by non-visualization, discontinuity, abnormal pathway, marked thinning, irregularity, or inhomogeneous signal increase. Ligament attenuation can occur due to ligament stretching caused by repeated injuries and failure of the self-healing process [30]. Ligament thickening may suggest some kind of reactive fibrotic change. Even in chronic sprain, the ligament may appear wavy or thick, and the signal intensity does not increase on proton- or T2-weighted images, leading to a false-negative diagnosis. Therefore, it is inevitable that the comprehensive evaluation of IOLV using only MRI has a limited role in estimating clinical instability.

According to a previous study, the IOLV in control group had a thickness of 2.2 mm and a width of 8.8 mm, and STI patients had IOLV with a thickness of 1.7 mm and a width of 7.2 mm [15]. The differential values between STI patients and controls have been reported as 2.1 mm thickness and 7.9 mm width. Another study involving patients with isolated LAI, whose IOLV was presumed to be intact, reported that the IOLV had a thickness of 2.1 mm and a width of 8.6 mm [3]. Conversely, patients diagnosed with isolated STI, whose IOLV was presumably torn, showed a thickness of 1.5 mm and a width of 7.3 mm. In the previous study, a cutoff of 1.8 mm thickness and 8 mm width was helpful in distinguishing between STI and LAI. In the present study, the intact IOLV group showed dimensions of 2.1 mm thickness and 7.9 mm width, and the group with IOLV tears showed dimensions of 1.9 mm thickness and 6.3 mm width. Although the difference in ligament thickness between the two groups was not significant, it was similar to previous studies showing that torn IOLVs were thinner and narrower in width. However, the IOLV tears carried a significantly smaller width than the intact IOLVs. ROC analysis of the IOLV width showed a cutoff of 7.50 mm to differentiate between torn and intact IOLVs. In contrast to previous studies in which the control group was only clinically diagnosed, the present study involved patients with arthroscopically confirmed IOLV status. Therefore, the clinical significance will increase as the MRI-surgical correlation is established.

As an ancillary finding, CFL injury was observed in 66.7% with isolated STI, 93.3% with STI and LAI combined, and 72.7% with isolated LAI. Overall 78.9% of patient with STI or LAI had CFL injury. CFL injury was common in both STI and LAI patients, suggesting that CFL may play an important role in the stability of the subtalar and tibiotalar joint as previously reported [3, 26].

Another point worth mentioning is that there was no difference in tarsal sinus tenderness and tarsal sinus fat abnormality between IOLV intact group and IOLV tear group. IOLV tear has been reported to be frequently associated with STI [3, 15]. Tarsal sinus tenderness and tarsal sinus fat obliteration are common findings in ankles with sinus tarsi syndrome [21, 22]. Accordingly, sinus tarsi syndrome may develop with or without STI. Therefore, it can be inferred that the presence of tarsal sinus tenderness and tarsal sinus fat abnormality do not suggest IOLV tear and cannot be an absolute criterion for diagnosing STI.

There are several limitations to this study. First, the retrospective nature of this study may have introduced biases in clinical information and radiological assessment. The correlation between clinical and imaging outcomes has not been fully evaluated due to the small sample size. Second, individual analyzes of ligament injury based on complete, partial, or intact ligaments are another limitation. We did not analyze the IOLV injury based on acute or chronic phases. In the chronic phase of ligament injury, the area of the tear is reshaped and filled with scar or fibrous tissue. It can be thick or wavy, making it difficult to detect the injury site and rate the injury grade. Third, only IOLV was evaluated in the subtalar structures. In contrast to IOLV, the CFL has yet to be routinely evaluated via open surgery or arthroscopy in cases of sinus tarsi syndrome without accompanying instability. Therefore, it was not possible to correlate MRI results with arthroscopic findings in these cases. IOLO, cervical ligament and inferior extensor retinaculum were not evaluated because of the difficult surgical correlation. Although osseous structures and tendons may also play a role in active stabilization of hind foot, this study focused only on the ligamentous structure as ligament reconstruction has recently been attempted to treat peritalar instability.

## Conclusions

Isotropic 3D MRI was feasible to evaluate IOLV in patients with clinically suspected STI and sinus tarsi syndrome. Addition of isotropic 3D sequences increased the diagnostic accuracy compared to conventional 2D sequences for the detection of IOLV tear. Further, isotropic 3D sequences may be more valuable for evaluating IOLV in cases of tarsal sinus fat abnormalities.

## Data Availability

Datasets used and/or analyzed in the current study are available from the corresponding author on reasonable request.

## Abbreviations

IOLV: Interosseous ligament vertical segment
STI: Subtalar instability
LAI: Lateral ankle instability
CFL: Calcaneofibular ligament
IOLO: Interosseous ligament oblique segment
TSE: Turbo spin-echo
AUC: Area under the curve
ROC: Receiver operating characteristic curve
